# A systems based qualitative analysis exploring the potential to implement risk stratified bowel cancer screening in England

**DOI:** 10.1186/s12913-025-12381-w

**Published:** 2025-02-11

**Authors:** Sowmiya Moorthie, Lily Taylor, Rebecca Dennison, Juliet Usher-Smith

**Affiliations:** 1 PHG Foundation, University of Cambridge, Cambridge, UK; 2 Cambridge Public Health, Interdisciplinary Research Centre, University of Cambridge, Cambridge, UK; 3 Primary Care Unit, Department of Public Health and Primary Care, University of Cambridge, Cambridge, UK

**Keywords:** Bowel cancer, Cancer screening, Risk stratification, Health policy, Risk prediction

## Abstract

**Background:**

Improving bowel cancer screening programmes through the introduction of risk stratification has been discussed, but not widely implemented in many countries. This study aimed to gain an understanding of system and programmatic aspects that need to be addressed to enable a move towards implementation.

**Methods:**

The Engineering Better Care Framework was used to support exploration and thematic analysis of the views of stakeholders involved in delivery of bowel cancer screening in England. Semi-structured interviews (*n* = 11) were conducted to gain a better understanding of the problem, and to identify elements that would contribute to a well-designed programme and anticipate barriers to delivery.

**Results:**

There is enthusiasm for introducing risk stratification and it is considered to be beneficial to improving the current screening programme. A major barrier impacting implementation is a lack of consensus on the best approach for risk stratified screening. Many interviewees recognised this as a bottle-neck and were in favour of processes that would enable more joined up decision-making to enable balanced consideration of the differing, and often nuanced potential of different strategies for risk stratification.

Several key considerations and design elements were identified: evidence demonstrating benefit of a particular strategy, feasibility of programme delivery (data availability, workforce capacity, public and patient acceptability, impact on equity), as well as changes to design of patient communication materials, the bowel cancer screening system, consent and follow-up services.

**Conclusions:**

System level issues and clarification of remaining uncertainties require resolution in order to move towards implementation. Moving this agenda forward requires consensus across different stakeholders in the first instance on the best use of risk. This will enable outlining key outstanding evidence gaps and establishing evidence thresholds for implementation. There are opportunities to design an optimal system based on harnessing existing infrastructure and learnings from other screening programmes.

**Supplementary Information:**

The online version contains supplementary material available at 10.1186/s12913-025-12381-w.

## Background

Cancer is a leading cause of mortality and morbidity worldwide. In many countries organised population-based screening programmes are in place to reduce mortality and morbidity through early detection. The structure of such programmes varies globally and across cancer types. With the exception of those with very strong family histories or known pathogenic risk factors, screening programmes tend to operate a fixed regime based on age and/or sex that is often referred to as “one size fits all”. While there are recognised benefits of such programmes, there are also associated harms [1]. Risk stratified approaches to cancer screening are proposed as a mechanism to improve the benefits of screening whilst reducing the harms [2]. This is through providing a more personalised approach to screening, by taking into account a wider range of individual level factors, such as family history, lifestyle and/or genetics or the results of previous tests [2, 3]. Indeed, we have seen increases in targeted or more stratified approaches to cancer screening in England over recent years. For example, following the introduction of Human Papilloma Virus (HPV) testing as the primary mechanism for cervical cancer screening has meant that this result can also be used to determine screen interval [4, 5]. National roll out of a targeted lung cancer screening programme was announced in June 2023. This programme identifies eligible populations on the basis of their history of smoking and risk of developing lung cancer [6].

The current NHS bowel cancer screening programme in England involves completing a faecal immunochemical test (FIT) every two years; those with a positive result (defined as ≥ 120 µg/g faecal haemoglobin) are referred for a colonoscopy [7]. Men and women aged 54 to 74 years are eligible for screening, with the starting age being decreased to 50 years. There has been interest for many years in improving the current screening programme through the introduction of risk stratification [3]. Different approaches to risk assessment and stratification for bowel cancer have been discussed in the literature [8]. However, these approaches are yet to be implemented and discussion is ongoing about their relative merits [9]. Bowel cancer screening programmes can be considered complex interventions in that they require a series of components, including people, equipment, processes and providers across the health system to come together to deliver effective and efficient programmes. A move towards introducing stratification into such programmes will likely add to this complexity, especially if the aim is to increase tailoring of services. There is currently insufficient information as to the key pragmatic and policy barriers to such an approach, thus making it hard to judge the level of complexity that will ensue and the impact this will have on programmes as a whole. In particular, while there is evidence from modelling studies that stratified screening may be beneficial, it is not clear how risk stratification would be implemented or what the system and programmatic aspects of such an endeavour would be [2, 8, 10].

Implementation strategies for different approaches to stratification, (both the stage in the pathway and mechanism for stratification) are likely to vary, as they may give rise to different challenges, each with associated policy and practice implications. A better understanding of the system and programmatic implications of introducing risk stratification is needed in establishing key challenges and uncertainties in moving towards implementation.

A 2017 position document published by the UK Royal Academy of Engineering in partnership with the Royal College of Physicians and the Academy of Medical Sciences defined a systems approach to health and care improvement [11]. It also outlined the value that an engineering-informed systems approach can bring to healthcare. This approach applies a variety of methodologies to a common framework that can be used to explore stakeholder views and identify questions that need to be answered for improvement. This framework has been used by others to address a wide range of complex healthcare issues and there is evidence in support of using this to improve healthcare pathways [12]. The implementation of any form of risk stratification to existing bowel cancer screening programmes can be viewed through the lens of an improvement initiative and thus amenable to analysis through this framework.

In this paper we report a systems-based approach to explore implementation of risk stratification to the bowel cancer screening programme in England. The systems approach framework was used to explore the views of those involved in delivering the bowel cancer screening programme to identify the key gaps in moving towards a risk stratified approach. A better assessment of these will enable identification of actions that can support a move towards delivery of a stratified bowel cancer screening programme.

## Methods

We explored the views of a range of stakeholders involved in delivery of bowel cancer screening, supported by the systems approach framework [13]. In the first stage, a map of the current screening pathway was developed using publicly available resources, including government guidance [14, 15]. This map (Supplementary materials) was used to identify an initial list of stakeholder roles involved in programme delivery. An interview study was then conducted with stakeholders using the map as a basis to refine the pathway and gain a better understanding of the impact of introducing risk stratification at different points in the current pathway.

### Sampling and recruitment for the interview study

The study population were professionals involved in delivering the screening programme in England. We aimed to interview approximately eight individuals depending on recruitment, availability, and when sufficient depth of understanding with which to draw conclusions about the research was reached. An initial list of individuals was compiled on the basis of our pathway mapping and using our networks. In addition, snowballing was used to identify any other individuals to approach for participation. Participant eligibility was ascertained by personal communication prior to data collection. All potential participants were provided with details about the aims of the study and written informed consent for participation. The study was approved by the Humanities and Social Sciences Research Ethics Committee [HSSREC] at the University of Cambridge [HSS rec reference 22.314].

### Data collection and analysis

One round of semi-structured interviews was conducted online via videoconferencing, using a topic guide (Supplementary materials). The topic guide was designed taking into consideration the perspectives that are considered as part of the engineering systems approach - general improvement, people, systems, design and risk [13]. Questions were designed to develop a better understanding of the problem, and to identify elements that would contribute to a well-designed programme and barriers to delivery of such a programme. The interviews were led by a policy analyst and supported by a PhD student, both with previous experience in conducting interviews and other qualitative methods. In addition, the study team included an academic clinician and non-clinical researcher with previous experience of qualitative research and research around risk stratification within the bowel cancer screening programme more generally. Given the background of the team, we endeavoured not to share personal perspectives during the interviews.

Given differences in descriptions of stratification, we began interviews by asking participants about their understanding of stratification and key points where it could interact with the current pathway. The map of the current screening pathway (Supplementary materials) was used to guide discussions and identify points in the current pathway that would be impacted by introduction of three specific scenarios of stratification. These were risk stratification of individuals according to risk and based on data from previous attendance, where relevant, to determine:

- Who is invited to screening
- The screening test modality
- The screening interval

Data from the interviews were pseudonymized and transcribed prior to analysis. Codebook thematic analysis [16] was carried out using NVivo V.12 software as there were defined discussion points established within our initial interview schedule. Pilot coding was performed for two transcripts by two independent members of the research term (SM + LT). Coded data were discussed and reviewed in consensus meetings to establish a common framework that was applied to the remaining transcripts. The coded data was then reviewed to identify lower-level themes within the framework, based on codes that conveyed similar meaning and areas of convergence. The finding were then organised according to the different perspectives of the Engineering Better Care framework [11].

## Results

### Participants

A total of 11 individuals were interviewed as part of this study with a range of roles across delivery of bowel cancer screening. This included members of, or advisors for, the UK national screening committee (UKNSC), those directly involved in delivery of the bowel cancer screening programme, such as developing the specifications of the screening programme, commissioners or as part of the screening service (e.g. part of a screening hub, endoscopist), and those not directly involved in the screening service but impacted by it e.g. GPs. We also interviewed experts in risk stratified screening. Most participants had multiple roles. To reduce identifiability we have attributed quotes throughout the text to a participant number and type of role.

### Codebook thematic analysis

**Fig. 1:**
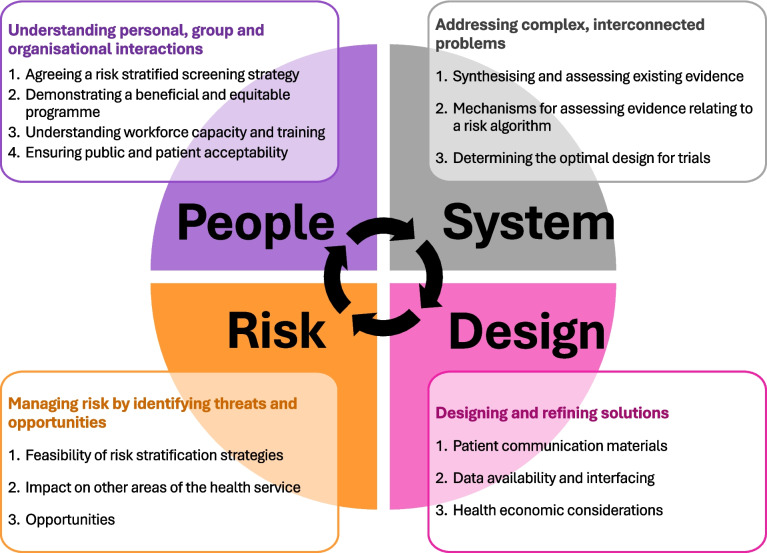
Summary of the findings according to different perspectives on risk stratified bowel cancer screening

The results of the interviews are summarised under themes which reflect the different perspectives (people, systems, design and risk) that are considered as part of the engineering systems design approach (Fig. 1). These perspectives contribute towards a better understanding of the problem, the elements to consider in delivering an improved system and what success of such a system would look like, including challenges and opportunities. Although many of the identified themes are subject to a degree of overlap, we describe them according to the most relevant systems perspective, whilst acknowledging that they may also apply to and impact upon other areas of the system (Fig. 1).

### People perspective

This perspective aims to gain a better understanding of the problem by assessing who will use the system, its current status and what affects it. Exploration of participants’ views on the current screening pathway, the concept of stratification and considerations in delivering a stratified screening programme informed this perspective. This allowed identification of five sub-themes covering over-arching issues that are impacting decision-making with regards to stratified bowel cancer screening that are included in Table 1 and described in more detail below.

**Table 1:** The people perspective – understanding personal, group and organisational interactions

Sub-theme	Illustrative quotes
Building consensus amongst stakeholders	“Or we have multiple people thinking they’re creating that information, which ends up with the scenario that you get multiple competing strategies. […….] we need one clear front runner, or a sequence of front runners, because otherwise you end up with the: which one is going to deliver the greatest benefit? and we never move out the starting block.” – P8 Screening delivery “Again, it’s all talking shops and nothing progresses. So, I think it needs a group… and I think by having people, for example someone from the hub… then if you have a clinical director, one of the colorectal surgeons who does the colonoscopies,[ ….].a charity, yeah, a patient representative[…].So yeah, I think a multidisciplinary group is probably key, because otherwise you don’t know how the system works.” – P7 Screening hub “You’d also need to decide the implementation strategy, big bang [or], phased. If you wanted phased, what’s the description of phase that you would need to implement? Is there to be any sign-off process at a hub or screening centre level? […] you’d have to build functionality to turn it on at different levels of granularity.” – P8 Screening delivery
Demonstrating a beneficial and equitable programme	“Yes, the main barriers would be does it impact on uptake, do you lose people?” – P3 Clinician “Is it going to widen inequalities? People who don’t access screening, is this going to actually mean that they’re less likely to access screening, [……] what extra work is this for the patient.” – P6 Clinician
Understanding of workforce capacity and training	“I think certainly capacity is a big issue […] but I think they [screening centres] are going to come up against in the next year real capacity challenges because each age group that comes on board is compounding the increases from the previous year.” – P11 Screening committee “The complexity there would be the availability of the screening centres. So currently, we have X number of slots in each screening centre for colonoscopy, so they only have to provide colonoscopy, or SSP slots and then colonoscopy.” – P7 Screening hub
Ensuring patient and public acceptability	“Politics, you mustn’t forget politics. So one of the things that happens is that there will need to be a consideration of those people who think they’ve been promised two-yearly and people like me [….] say you don’t need it for another six. Now they might not like that, politicians certainly might not like that and so in other circumstances there’s been a transition where people who currently have been promised something or other continue to get it, while the new people get the new thing.” – P1 Screening commissioning “Obviously if you are now making the change, even along the lines of gender [sex], and inviting women earlier than men, [……] but just even on the first invitation basis of who do you invite? That might still be controversial from a public acceptability point of view.” – P2 Researcher

#### Building consensus amongst stakeholders

All participants, apart from one, were aware of the concept of stratified screening, with some participants (*n* = 4) considering it as already being part of the current programme. This was because individuals who enter the programme are already selected on the basis of age, and follow-up for individuals who undergo a screening test can differ. For example, a proportion with a positive screening test may be offered CT-colonoscopy (CTC) as opposed to colonoscopy. It was also stated that those with a family history or who are at high-risk due to high penetrance genetic variants are offered different screening options from those in the general population. In addition, Lynch syndrome testing has been aligned with the bowel screening programme. Therefore, the programme was felt to already incorporate risk stratification based on age and, to a degree, family history.

A move towards increased stratification was viewed favourably, however many stated that an agreement on the shape of such a programme is yet to be reached. Participants reflected on the fact that multiple potential strategies exist for bowel cancer screening, from the point on the pathway at which it is introduced to the types of data included in a risk algorithm and even the desired outcomes. Selecting one or more favourable approaches was seen as an important step to move beyond talking about the options to actually implementing risk stratification.

Several participants identified the need for a consensus decision-making approach to achieve this. This was particularly important for some who reflected that the current approach to decision making is somewhat siloed (Table 1) For them, this segregated approach to decision-making represents a significant barrier to achieving the joined-up thinking required to make concrete decisions as to the shape of a risk stratified programme. Interviewees also stipulated the need for involving a spectrum of stakeholders in any consensus building efforts. For example, by involving the members of the UKNSC who assess the evidence base, those involved in developing the specifications of the screening programme and researchers may enable achieving consensus (Table 1).

Participants stated their belief that achieving consensus on the specific risk stratified screening strategy will influence decisions regarding the way in which implementation is to be rolled out. This could be a phased or incremental implementation in which more straightforward strategies using data within the current system (age, sex and faecal immunochemical test [FIT] result) inform risk stratification and are implemented as phase one, working towards use of increasingly complex risk models requiring additional data in subsequent phases. Alternatively, as changes to the Bowel Cancer Screening System (BCSS), the digital system that supports delivery of the programme, are challenging, some could see an argument for implementing more data-intensive risk stratification (including novel data points) in a single step.

#### Demonstrating a beneficial and equitable programme

A further consideration that participants felt impacts on choosing a strategy is that screening programmes aim to be delivered to a consistent standard across the population. Ensuring that introduction of any form of risk stratification does not exacerbate inequalities in screening is a key concern. The current pool of people who are invited for bowel cancer screening are identified from GP registers and a proportion will decline screening. This was felt likely to be the case going forward with the introduction of any form of stratified screening. Thus, participants expressed the need to balance improvements of services for those who already take-up services with improving services to ensure wider uptake and therefore wider benefit.

The discussions also re-iterated that decisions in relation to screening policy and development of screening specifications are complex endeavours. This is because whilst the general consensus is that bowel cancer screening is beneficial, similar to other cancer early detection and screening programmes, demonstration of benefit is not straightforward. These will continue to be issues even with introduction of stratified screening.

#### Understanding workforce capacity and training

Workforce training and capacity issues were discussed by eight of the 11 interview participants. Introduction of any form of risk stratification would require training of staff across the screening programme to enable a better understanding of changes to the programme. The extent of training needs will be dependent on the level of changes made.

The most salient concern was capacity of colonoscopy resources, including screening endoscopists, and availability within screening centres. Although one of the aims of risk stratification is to aid in alleviating such pressures by distributing colonoscopy resources more efficiently, several participants expressed apprehension that this issue may in fact be intensified by changes to the screening programme, particularly if high risk individuals were offered intensified screening without also de-intensifying screening for low risk groups. However, one participant reflected that the screening programme already has an element of flexibility built in to accommodate fluctuations in resources and invitation rates and speculated that this might help to prevent screening hubs from becoming overwhelmed.

Additionally, the extent of involvement of primary care was a particular issue for consideration in relation to invitation to screening. Primary care professionals are not currently directly involved in population screening programmes for bowel cancer. If implementation of risk assessment requires primary care to either support data collection or discuss results, this would have an impact on training needs and capacity as explained under the ‘risk perspective’.

#### Ensuring public and patient acceptability

Several interviewees identified that risk stratified bowel screening must be acceptable to both patients and the public, and one participant also introduced political acceptability as a potential barrier or facilitator of wider acceptability.

Participants anticipated that treating certain groups of society differently to others could be controversial or even discriminatory in the eyes of the public, emphasising the need for robust evidence to justify the shift to risk stratification. There were concerns that while de-intensifying screening for low risk individual is a possibility it may not be acceptable to the public. Some also speculated that low public acceptability may have negative ramifications for uptake or that people may simply request FIT kits from primary care to circumvent the new system. Ultimately, public acceptability appeared to depend on a successful approach to public communication.

### Systems perspective

This perspective considers the interplay between technical and social factors within a complex system that result in specific properties or behaviours. It aims to identify particular problems that require solutions and explore options in developing solutions. We grouped under this theme specific problems that were raised by participants that impacted on decision making. Three key problems were raised: 1) synthesising and assessing existing evidence to inform selection of a risk stratification strategy, 2) mechanisms for assessing evidence relating to risk algorithms and 3) trialling the optimum risk stratification strategy (Table 2).

**Table 2:** Systems perspective – addressing complex, interconnected problems

Sub-theme	Illustrative quotes
Synthesising and assessing existing evidence	“Okay, so in an ideal world, we’d have a full screening RCT that follows up people from the point of being invited to mortality. The reality, the feasibility to do that kind of a trial you need to be really… you need to have narrowed down and done a lot of pre-work to understand which risk stratification approach you’re going to evaluate so there’s a lot of work before you get to that stage.” – P9 Screening committee
Mechanisms for assessing evidence in relation to a particular risk algorithm	“If you’re thinking about, for example, polygenic risk scores, yes, they could be incorporated within the decision support [risk algorithm] but there’s still quite a long way to go yet in terms of proving the validity [….] and their utility based on evidence. It depends on what level of evidence you are happy to rest on before you start to implement a new stratification tool.” – P5 *(Clinician)* “There’s different stages to that, isn’t there, because there’s the scientific validity to start with which is obviously independent of any IT, and what’s the minimum data set that you need to make it scientifically – to meet the scientific validity and then there’s in terms of actually using it in practice, making sure that you’re building a tool that’s going to be at least interoperable if not embedded and picks up all the relevant information.” – P6 Clinician
Determining optimal design for trials	“We then also need to have determined the success criteria, [….]. You might have been working your requirements from an IT point of view, but you won’t have committed to build unless somebody’s willing to go at risk that somebody is going to approve that that is actually able to be implemented.” – P8 Screening delivery “The clinical evidence is obviously borne out of your trials, so I suppose that’s where that becomes difficult. Do we already hold data that would provide the evidence already? Or do you need to run a real-time clinical trial to be able to do that? And then the question becomes, is it appropriate to conduct that trial with your screening population? Or is it more appropriate to conduct that in a symptomatic population and extrapolate your outcomes? I think it depends on the question you’re seeking to answer.” – P8 Screening delivery

#### Synthesising and assessing existing evidence

As noted earlier, several participants stated that many options are available in risk stratified screening, however, it is unclear which of these options is “best”. Participants acknowledged that ideally a clinical trial would be used to assess the feasibility of a risk stratified bowel cancer screening programme, but highlighted several outstanding questions relating to the volume and quality of evidence needed to move forward and the most appropriate population to sample from. Once again, the need to define a clear strategy emerged as a priority. This was needed to develop an understanding of what to evaluate and key outcome measures. Therefore, evidence synthesis is needed to inform how existing and ongoing work on bowel cancer risk prediction informs a screening programme and identification of optimal strategies that are ready for implementation and could be incorporated into a trial.

#### Mechanisms for assessing evidence relating to a risk algorithm

Many participants stated that validating and assessing a risk algorithm or the use of particular data points as part of risk stratified screening was an essential step in moving towards implementation of risk stratification. However, gaps in knowledge on how to approach evidence appraisal in relation to risk algorithms and acceptable levels of evidence were cited as issues.

#### Determining the optimal design for trials

The need to evaluate and monitor the outcomes of a particular risk stratified screening strategy were highlighted as important early considerations to avoid complications in the long term and to ultimately enable evaluation of the success of the programme. This included practical consideration of ensuring that any risk stratification mechanisms developed are compatible with the wider system. Similarly, the desired outcomes for a risk stratified bowel screening programme need to be clearly established a priori in order to enable effective monitoring and evaluation of such a programme.

### Design perspective

The ‘design perspective’ primarily aims to identify which design challenges need to be solved and to operationalise this by refining the optimum solution to deliver outcomes as intended. To enable discussion around particular design needs, the interviews were structured to focus on three scenarios of risk stratified screening. From the conversations it became apparent that delivering risk stratified screening required consideration of common aspects across all three scenarios. These included patient communication materials, consent, the specification of the electronic data system - the BCSS, and health economic evidence (Table 3).

**Table 3:** The design perspective – designing and refining solutions

Sub-theme	Illustrative quotes
Patient communication materials	“I think it is possible that people will sort of assume the worst when these changes happen unless they are very well explained to them. They will immediately think that somebody has made a mistake, or somebody is trying to deprive them of something. So, it is quite easy to politicise that change even at an individual level.” – P2 Researcher “Yes, I’m sure that we communicate to patients would have to change. And I suppose the risk here or the concern would be that as soon as you start to talk about lower risk then actually you start to get much lower participation rates.” – P3 Clinician
Data availability and interfacing	“When we then look at the data quality, it has been poor, and that’s because when the data were captured, they were captured for the purposes of disease registration, not for the purposes of enabling an invitation to a screening programme… We ask people questions, and we ask them in good faith, yes, of course those data exist, absolutely they exist. Do they exist at the level of accuracy and granularity required to mobilise that information to invite people for screening? No.” – P8 Screening delivery “It’s about the minimum data set really that’s needed for the risk tool and how that data is collected and who collects those. Whether it’s collected in primary care, whether it actually has to be collected by a healthcare professional or whether it’s just the information comes in and it’s coded. Again, the more automated, the better really. So, although I’ve spoken about the time it takes to file results… if we just had a printout that said weight, height, polygenic whatever, then data is inputted. It’s still a point in the process but it’s quicker than somebody in primary care actually doing it…” – P6 Clinician
Health economic considerations	“It’s this invest to save philosophy as well, that you might not necessarily see benefits instantaneously […] it could be something we have to understand that we need to have more money not less initially to be able to do something […]” – P8 Screening delivery

#### Patient communication materials

Updated patient communication materials would be required with any change to the programme. The differences between the potential risk stratification scenarios were in relation to the extent of changes that would be required, as these are likely to vary depending on the shape of the screening programme. For example, changes to eligibility, especially using additional phenotypic or genetic data would require substantial changes to the current information leaflets. Two interviewees further considered the communication of risk stratified screening features and personalised risk estimates. Both agreed that this was an important design feature and also highlighted several pitfalls that could occur if public communication is poor, specifically reduced uptake.

Conversely, one individual also went on to suggest that the communication of risk and stratified screening could present an opportunity to improve uptake whilst simultaneously narrowing inequalities, and could also be used as a teachable moment for the introduction of behaviour change interventions.

#### Data availability and interfacing

Whilst the validation of a risk algorithm is considered within the ‘systems perspective’, the ‘design perspective’ is concerned with the availability and collection of the required data. Many participants noted that primary care records hold less data than one might expect, such data is challenging to obtain and oftentimes the quality is poor. The availability of good quality data that can feed into specific algorithms is a key consideration for any risk stratified programme.

The minimum data set required will be dependent on both the chosen risk stratification strategy and the risk algorithm. A further consideration is how data could be collected, whether that would require a healthcare professional or if self-reported data is sufficient, or whether it is possible to collect certain novel data such as polygenic risk information alongside the FIT kit. Participants were sceptical about the extent to which primary care professionals may be capable of collecting or providing the relevant data, suggesting that data may be best obtained directly from screening programme participants. They also emphasised that “the more automated, the better”, suggesting that the method of collecting and inputting data must be appropriately interfaced with the BCSS to mitigate the impact on healthcare professionals. It may then be the case that additional support would be needed in order to ensure data availability and make changes to the BCSS.

#### Health economic considerations

One participant stressed that cost was an important factor when considering programme viability and again, recognised that this will vary depending on the chosen strategy as well as over time. The concept of frontloading the cost was posited in order to make financial gains in the long term.

### Risk perspective

This perspective aims to examine potential challenges and opportunities to particular solutions. This was explored by discussing challenges and opportunities to delivering a risk stratified bowel cancer programme, and any research or policy gaps that need to be addressed. This perspective also included possible risks to the system, for instance, overall feasibility and any unintended consequences that risk stratification could have for other areas of the health service (Table 4).

**Table 4:** Risk perspective – managing risks by identifying threats and opportunities

Sub-theme	Illustrative quotes
Feasibility of risk stratification strategies	“And I think that one of the challenges is hitting that sweet spot between being able to do in the screening programme, because we're very structured and we're very methodical, which gives you the ability to be able to capture that data, capture those outcomes. But that comes at the expense of needing to develop all of that functionality, and also growing your workforce to be able to support your trial which has a lead-in time.” – P2 Researcher “So, if it’s things [data] that we already have available from the GP systems, which is very little actually, so it’s age, sex and their postcode gives us their IMD status, but we don’t even know if people have got a history of cancer. We don’t know if they’ve got a special need […] so, it’s very basic, the system, as it is currently. So, I think in terms of what’s required on the system, that would then require some downloading from GP systems and that is quite complex.” – P7 Screening hub
Impact on other areas of the health service	“So, we are in a resource constrained system, so if we are then potentially increasing the number of tests, whether it’s solely FIT tests, whether it’s poo in the post, whether it’s sigmoidoscopies, what’s the impact going to be on the wider system there? Certainly, that could have an impact on general practice again.” – P6 Clinician “So, for example, we have colleagues that support us obviously with pathology. Now, obviously you might have a different impact in pathology dependent upon your risk stratification, you also might have depending on what you stratify and what type of diagnostic test.” – P8 Screening delivery
Opportunities	“So the bowel cancer screening IT is amongst the most modern… And so it should be capable of assigning people different follow ups…” – P1 Screening committee “What Lynch has done is it’s added something different, and it’s been very interesting because there was this perception it was going to be very easy, and clearly it hasn’t been quite as easy as everybody thought. But then from that comes learning, so you would imagine that that would make it [risk stratification] more straightforward or, you know, people have learnt.” – P7 Screening hub

#### Feasibility of risk stratification strategies

Within this study we discussed three options of potential stages in the screening pathway where risk stratification could be introduced into the programme (at the point of invitation, the choice of screening test and the determining the screening interval). Views varied as to which and whether any of these options was most suitable for implementation. In general, participants were unclear which use of stratification would be most feasible currently. Introduction of any of the proposed strategies required balancing the “best” mechanism for stratification with feasibility of obtaining data to enable this. At present it was unclear what the balance between benefit and feasibility were for different options. This was also creating challenges for assessing specific risks and opportunities posed by different options.

#### Impact on other areas of the health service

Multiple interviewees considered the potential for unintended consequences of introducing risk stratification on other areas of the health service. Most notable was the impact on radiology resources, which are already constrained, if different screening modalities such as CTC are offered. Primary care, specialist screening practitioners (SSPs), pathology and IT departments all also had potential to experience an increase in demand as a result of risk stratified bowel screening. Whilst this challenge was not perceived as insoluble, it was highlighted as an integral component of achieving whole system readiness.

#### Opportunities

There was a general sense that the introduction of risk stratification could be beneficial to the bowel cancer screening programme. The fact that the bowel cancer IT is amongst the most modern was stated as an opportunity, as it has the capability of adapting to enable some form of stratified screening and was setup to incorporate a risk algorithm when FIT was introduced in 2019. This was in anticipation of potentially offering different screening intervals on the basis the result of a FIT test.

In addition, as the health system is already implementing more targeted screening programmes (e.g. lung cancer, Lynch syndrome), this provides an opportunity to learn and inform the design of effective systems for bowel cancer screening. Furthermore, one participant highlighted that the fact that the bowel cancer programme is already implemented means there is infrastructure in place for data collection on outcomes and monitoring of programmatic aspects. This provides an opportunity to conduct research that can answer some existing uncertainties in relation to quality improvement of the programme.

## Discussion

### Principle findings

Taking a systems approach has allowed us to explore and develop an understanding of core challenges to address in moving towards risk stratified screening for bowel cancer in England. Through this, we have shown that there is enthusiasm for introducing risk stratification and that it is considered to be beneficial through enabling improvements to the current screening programme. However, there is no consensus on the best approach for risk stratified screening amongst those commissioning and delivering the programme, which is a major barrier to moving forward with implementation. One of the drivers of this appears to be that decisions in relation to approval of/or changes to the programme, development of screening programme specifications and commissioning often occur separately. Given that selection of particular strategies for risk stratification requires a balance between evidence of benefit and feasibility of delivery, decisions with respect to what changes to implement cannot be made in siloes. Selection of an approach to implementation requires joined-up decision making to enable balancing the differing, and often nuanced potential of different strategies for risk stratification. Many interviewees recognised this as a bottle-neck and were in favour of processes that would enable more joined up decision-making.

The lack of consensus on what changes could/should be implemented has further created uncertainty in understanding the feasibility of programme delivery and therefore design of a programme. Drivers of lack of consensus include gaps in knowledge on how to approach evidence assessment in relation to a risk algorithm and the need to bring together and assess existing evidence in support of particular risk stratification strategies. Challenges around ensuring data availability and quality for risk assessment, workforce to implement changes to the IT system and the ability to manage the change process with limited resources were also reported as issues that would need to be addressed.

We were, additionally, able to identify key factors that would influence the shape of any programme. These were considerations around evidence demonstrating benefit of a particular strategy, evidence in support of a risk stratification algorithm (if one is used), evidence from health economic modelling, and feasibility of programme delivery (data availability, workforce capacity, public and patient acceptability). In addition, we identified key design elements that would be impacted by changes to the current programme. These were patient communication materials, bowel cancer screening system, consent and follow-up services.

Furthermore, our participants highlighted that the introduction of risk stratified screening in different forms did not always require consideration of novel factors. This meant that learnings from implementation of risk stratified approaches in other contexts such as lung cancer screening could be applied to the context of bowel cancer screening, albeit with the details and extent of change likely to vary across different scenarios of risk stratification.

### Strengths and limitations

Strengths of this study include the use of purposive sampling and a semi-structured interview guide. Purposive sampling allowed us to include participants with different roles across the screening pathway. However, the study was conducted with a limited number of health care professionals and many were aware of risk stratification. Thus, there is a possibility that we have not captured or reflected a full spectrum of views. The use of semi-structured interviews and open-ended questions allowed us to gather rich data, by enabling participants to speak openly.

To ensure consistency during analysis, all transcripts were double coded. Interpretation of the findings was discussed with the wider research team to encompass a range of perspectives. A further key strength of our approach was the use of the Engineering Better Health framework, allowing us to take a quality improvement approach and extract key themes. Using this framework has also allowed us to explore both overarching as well as specific opportunities and challenges for implementation of risk stratified bowel cancer screening, as well as outline key designs considerations for any form of risk stratification. Although there was some overlap of themes between the system perspectives as described, it provided a useful way of categorising the inter-related influences on introduction of risk stratified screening. The research team had previously knowledge of bowel cancer screening and risk stratification, which could bias findings. We endeavoured not to share our personal perspectives during the interviews and thefindings of the study were additionally validated by sharing with interviewees to ensure we did not misrepresent any views. Furthermore, our interviewees were based in England and our analysis was conducted from the perspective of the bowel cancer screening programme in England. This will impact on the transferability of findings to other countries and health systems. However, some issues such as in relation to design elements may be applicable to consider across countries and settings.

### Implications for policy, practice and research

The move towards risk stratified bowel cancer screening programmes is of interest in many high-income settings. Indeed, trials are underway in some settings [17, 18]. Many papers have been published discussing the options for stratified screening, performance and validation of risk prediction models and exploring patient and health professional views of stratified screening [9]. To our knowledge this is the first paper that has examined the challenges in delivering risk stratified programmes from a whole system perspective. This has allowed us to explore another dimension to the current literature and complement ongoing work that examines public and patient views [19–21] by identifying system level issues as well as particular questions that require resolution in order to move forwards towards implementation. It has also allowed us to distil a series of actions (Table 5) that can move this agenda forward. From a policy perspective, consensus decision making on the best use of stratification as part of bowel cancer screening pathways in the short, medium and long term is needed. This will require considering and balancing the options for incremental versus larger changes to the programme, the benefits and feasibility of delivery of different strategies for stratification and current research and implementation gaps. Current research gaps to address this process relate to evidence synthesis that enables comparison of different strategies for risk stratification and mechanisms to assess evidence in relation to a risk algorithm. Filling these gaps will enable identification of options that are feasible to trial in the short term.

**Table 5:** Actions that to facilitate implementation based on findings of the systems analysis

Perspective	Action
People	• Create opportunities to bring stakeholders together to reach a consensus on the best use of risk stratification in the short, medium and long-term. • Ensure that any chosen risk stratification strategy considers the current capacity and resource constraints and does not increase resource use overall. • Ensure that any chosen strategy does not put additional pressure on primary care. • Intentionally design communication strategies to enable evidence-informed public and political support. • Conduct additional research and impact assessments to explore the potential impact on underserved and screening averse populations and ensure that introduction of risk stratification does not widen inequalities.
Systems	• Outline key outstanding evidence gaps that will impact implementation. • Agree a mechanism to assess evidence in relation to a risk algorithm. • Establish the threshold of evidence needed for implementation.
Design	• Co-design public communication materials to facilitate understanding and acceptability. • Support development of an IT system that is able to operationalise risk algorithms and to adapt and evolve to future evolutions if risk stratification. • Design data collection to be as automated as possible to avoid additional strain on healthcare services. • Harness current screening and data infrastructures to enable monitoring of programme and outcomes. • Appreciate that upfront funding may be required to enable longer-term savings.
Risk	• Ensure that the potential impact on wider healthcare services (radiology, pathology, primary care, SSPs) is considered. • Conduct studies to test feasibility of particular uses of stratification, particularly around the feasibility of different data. • Build on learnings from implementation of risk stratification as part of other screening programmes to inform and shape bowel cancer screening. • Take advantage of the current bowel cancer screening IT system.

## Supplementary Information

Supplementary Material 1.

Supplementary Material 2.

## Data Availability

The pseudo-anonymised interview transcripts are stored at the University of Cambridge data repository (https://www.repository.cam.ac.uk/). Formal requests for access will be considered via a data‐sharing agreement that indicates the criteria for data access and conditions for research use and will incorporate privacy and confidentiality standards to ensure data security.

## Abbreviations

BCSS: Bowel Cancer Screening System
CTC: CT-colonoscopy
FIT: Faecal Immunochemical Test
GP: General Practitioner
HPV: Human Papilloma Virus
SSP: Specialist Screening Practitioner
UKNSC: UK National Screening Committee
