# Supplementary material for: A systems based qualitative analysis exploring the potential to implement risk stratified bowel cancer screening in England
Source: BMC Health Serv Res. 2025 Feb 11;25:226. doi: 10.1186/s12913-025-12381-w (PMC11812230; doi:10.1186/s12913-025-12381-w)
Supplement: Supplementary file 2 — Supplementary Material 2. [file 12913_2025_12381_MOESM2_ESM.docx]

**Systems based analysis of colorectal cancer screening pathway**

Topic Guide

This is a basic topic guide for semi-structured interviews with professionals working to deliver the bowel cancer screening programme in England. It will be adjusted as the study evolves. Questions will be tailored to individual participants and their expertise, and adapted for use with individual interviews.

1. Interviewee background

Participant’s professional role and involvement in the screening pathway.

Participant’s knowledge of risk stratified bowel cancer screening

- Your role in the bowel cancer screening programme
- How you interact with other parts of the screening pathway
- Your awareness of the use of risk stratification at the stage of the pathway that you work at

1. Screening programme map

- Elicit thoughts on our map of the current screening pathway, has any key information been missed or useful to discuss in the context of stratification (provided in the interview)
- How you think the current programme might need to change to implement risk stratification for these different uses:

Risk stratify individuals and determine who is invited to screening

Determine the screening test modality

Determine the screening interval according to risk and based on data from previous attendance.

1. Discussion on risk stratified screening

- We have identified requirements for delivery of risk stratified screening. Are there any that you disagree with or that we have missed.
- What challenges need to be addressed (from your specific perspective/role) for the implementation of risk-stratification for each of these purposes.

What opportunities are there for delivery of risk stratified screening
