# Supplementary material for: A systems based qualitative analysis exploring the potential to implement risk stratified bowel cancer screening in England
Source: BMC Health Serv Res. 2025 Feb 11;25:226. doi: 10.1186/s12913-025-12381-w (PMC11812230; doi:10.1186/s12913-025-12381-w)
Supplement: Supplementary file 2 [file 12913_2025_12381_MOESM1_ESM.docx]

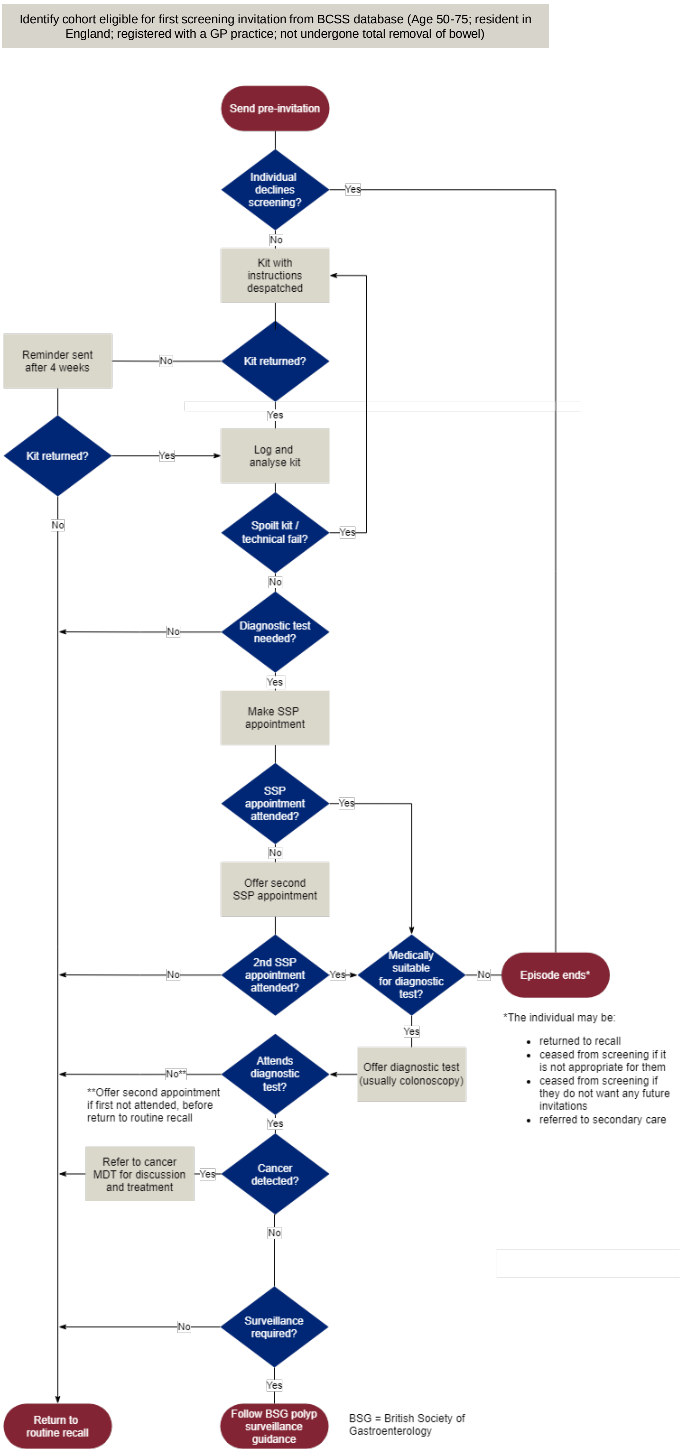

Adapted from: https://www.gov.uk/government/publications/bowel-cancer-screening-pathway-requirements-specification/bowel-cancer-screening-pathway-requirements-specification
